# Efficient Construction and Effective Screening of Synthetic Domain Antibody Libraries

**DOI:** 10.3390/mps2010017

**Published:** 2019-02-14

**Authors:** Arghavan Solemani Zadeh, Alissa Grässer, Heiko Dinter, Maximilian Hermes, Katharina Schindowski

**Affiliations:** 1 Institute for Applied Biotechnology, Biberach University of Applied Science, Hubertus-Liebrecht-Strasse 35, 88400 Biberach, Germany; arghavan.soleimani@uni-ulm.de (A.S.Z.); Alissa.graesser@t-online.de (A.G.); heiko.dinter@web.de (H.D.); MaximilianAlexander.Hermes@hochschule-bc.de (M.H.); 2 Faculty of Medicine, Graduate School “Molecular Medicine”, University of Ulm, Albert-Einstein-Allee 11, 89081 Ulm, Germany; 3 Faculty of Natural Sciences, University of Ulm, Albert-Einstein-Allee 11, 89081 Ulm, Germany

**Keywords:** display technology, antibody engineering, synthetic antibody library, shark antibody, vNAR, phage display, panning

## Abstract

Phage display is a powerful technique for drug discovery in biomedical research in particular for antibody libraries. But, several technical challenges are associated with the selection process. For instance, during the panning step, the successful elution of the phages bound to the antigen is critical in order to avoid losing the most promising binders. Here, we present an efficient protocol to establish, screen and select synthetic libraries of domain antibodies using phage display. We do not only present suitable solutions to the above-mentioned challenges to improve elution by 50-fold, but we also present a step by step in-depth protocol with miniaturized volumes and optimized procedures to save material, costs and time for a successful phage display with domain antibodies. Hence, this protocol improves the selection process for an efficient handling process. The here presented library is based on the variable domain (vNAR) of the naturally occurring novel antibody receptor (IgNAR) from cartilage fishes. Diversity was introduced in the Complementarity-Determining Region 3 (CDR3) of the antigen-binding site with different composition and length.

## 1. Introduction

Antibodies in particular immunoglobulin G (IgGs) are some of the most important biopharmaceutical molecules with a highly relevant market volume. Antibodies or other scaffolds have broad and diverse applications for the therapy of several diseases since they can bind almost any pharmaceutical target. To develop suitable therapeutic antibodies an efficient screening and selection technology is phage display, which allows the screening of large libraries [1,2].

Antibody phage libraries have become practical tools for the generation of monoclonal antibodies (mAb), antibody fragments like single-chain variable fragment (ScFv), antigen-binding fragments (Fab) or recombinant single-domain antibodies from camel (VHH) or shark (vNAR). The success of the selection process depends on a variety of elements including the quality and diversity of the initial library. The initial library can be either of a fully synthetic, semi-synthetic, naïve (non-immunized) or immunized (antigen-specific) origin [3,4,5,6]. However, sometimes antibodies selected from synthetic, semi-synthetic or naïve libraries show lower affinities for their antigen than antibodies selected from libraries of immunized animals, in which multiple rounds of immunization with the antigen were performed. This problem can be overcome by further refinement through in vitro affinity maturation using site-directed mutagenesis or error-prone Polymerase Chain Reaction (PCR).

Technically, construction of synthetic antibody libraries has the advantage of simplicity compared to the process of immunizing animals, amplifying their B-cells’ variable Ig sequences and, finally, introducing them into a phagmid vector for the phage display. Since this type of library obviates the need for animal immunization and allows selection of antibodies against many antigens including auto-antigens. Synthetic antibody libraries are constructed by introduction of degenerated nucleotides into the complementarity-determining regions (CDR) [4]. It is important to choose the appropriate framework (FR) in order to introduce diversity in the CDRs. Framework for synthetic libraries can be selected based on properties such as stability and expression of the antibody into *E. coli* (*Escherichia coli*) when phage display will be used as a technique for screening. The variable domain of shark antibody has four (hyper) variable loops: CDR1, hypervariable loop 2 (HV2), hypervariable loop 4 (HV4) and CDR3. Several studies have shown that the diversity of antibodies belongs predominantly to CDR3 loops which are tolerant to sequence and length variations [7,8].

Although methods for screening and selecting antigen-specific antibody fragments have become much more accessible, techniques for high-throughput selection with high yield, low cost and low effort are still required. Phage display is a widely used method to screen libraries. The physical linkage between the genotype and phenotype is the main strength of phage display. Therefore, filamentous phages express a foreign protein fused to a phage coat protein, i.e., pIII, displayed on their surface (phenotype). The DNA sequence of the foreign protein is cloned in-frame in front of the gene encoding the phage coat protein (genotype). The phage library is used to select and isolate binders with high affinities to the target antigen. The selection process is called biopanning [9,10]. Biopanning involves four main steps [11,12]. First, the displayed phage library is incubated on the antigen immobilized on a surface. After incubation, unbound and low-affinity binders are washed away while binders with higher affinities remain bound to the immobilized antigen. In the elution step, bound phages are eluted by pH shift or enzymatic treatment and used to infect *E. coli* cells for amplification. Typically, three to five rounds of biopanning are performed to enrich specifically binding phage particles. With each round of panning, the stringency of the washing steps increases and thereby the affinity of the binders improves.

Even though construction and screening of synthetic libraries by phage display technology has been widely used, several challenging problems can occur:

1. By introducing randomly selected nucleotides as a cost-efficient method during the construction of the synthetic library, stop codons can occur that significantly decrease its quality by lowering the number of clones expressing a full-length protein.
2. The stronger the binders, the harder it is to elute them from their antigen and, hence, the best binders can be easily lost during the selection process.
3. A significant bottleneck of a phage display selection is the production of sufficient amounts of bioactive monoclonal binders since the low expression level of properly folded proteins from the periplasmic space can be challenging.

In this protocol, we describe a simple method for the construction of a synthetic vNAR library with codon-wise mutagenesis by using degenerated NNK codons (N means a 25% mix each of adenine, thymine, cytosine and guanine nucleotides; and K stands for a 50% mix each of thymine and guanine nucleotides). The probability of introducing a stop codon exceeds 50% after using ten continuous NNN codons, while this will only happen after sixteen codons in case of using NNK codons. The NNK degenerated codons code for all 20 amino acids and only for the amber stop codon (TAG or amber codon) while NNN primers code for all three stop codons [4,13]. The TAG stop codon can be translated to glutamine in *E. coli* strains with a glutamine-inserting amber (UAG) suppressor tRNA (*SupE*), such as TG1. Hence, using TG1 can abolish the effect of introducing stop codons. Furthermore, using NNK primers decreases codon redundancy, i.e., arginine encoded with six codons is reduced to three possible codons. Hence, this design increases the possibility of introducing amino acids like tryptophan, which are encoded by one codon only. The advantage of such a design is that the oligonucleotide can be easily synthesized at low costs [14] compared to other methods avoiding stop codons [15,16]. 

Another challenging step in the phage display process is the elution of bound phages during biopanning. In general, one of these three methods are used to either reduce the antigen-binder interaction or to cleave the phages from their displayed binder protein: (1) low pH [17], (2) high pH [18], or (3) enzymatic cleavage by trypsin. For this protocol, a phagemid was chosen, which possesses a cleavage site for trypsin. However, since the standard procedure for the trypsin digest was insufficient (see Table 2), we have improved the elution process by applying trypsin in two steps. By using these measures, we could improve the elution by 50-fold.

Sufficient production of monoclonal binders in a 96-well plate is highly critical for phage display selection. Because of the small volume of medium in the wells of a 96-well plate, it is difficult to express them in a sufficient amount. Furthermore, expression of immunoglobulin-based binding proteins in the periplasmic space can result in partially unfolded or aggregated species [19] and such proteins exhibit a decreased bioactivity in the enzyme-linked immunosorbent assay (ELISA) [20]. Therefore, we have implemented a tailored periplasmic buffer, which results in an improved release of proteins from the periplasmic space. The increased amount of bioactive binding protein results in higher signals in the monoclonal ELISA. 

## 2. Experimental Design

The vNAR antibody scaffold used for synthetic shark antibody library is based on HEL-5A7, a type I vNAR produced by Helen Dooley from an immunized library against lysozyme [21]. This template was used by Shao et al. to construct a synthetic vNAR library [22]. The framework was chosen due to its high stability and functional expression in prokaryotic systems. It was synthesized with flanking *Nco*I and *Not*I restriction sites at its 5’ and 3’ end, respectively, for subsequent cloning into a pSEX81 phagemid vector. 

**Figure 1:**
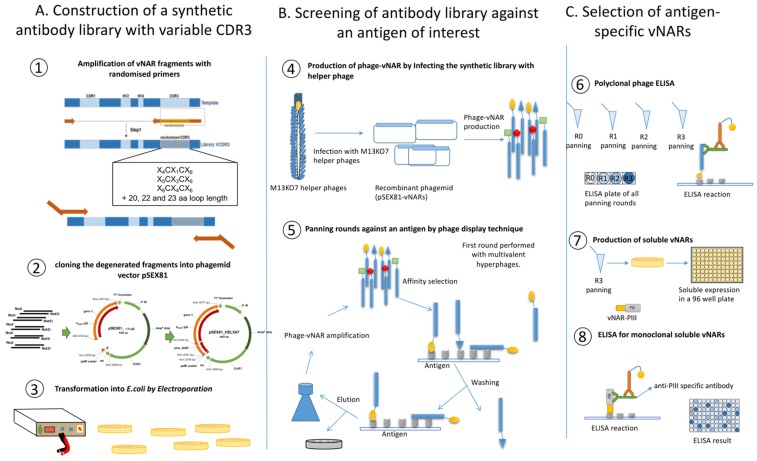
Schematic representation of the improved protocol.

Sequence diversity is confined to the CDR3 region. Completely randomized sequences are used with different lengths (Table 1) and with two cysteines in conserved positions, corresponding to a type I vNAR. CDR3 diversification is achieved in a two-step splicing by overlap extension polymerase chain reaction (OE-PCR; Figure 1). Diversity in CDR3 is introduced by using randomized primer and HEL-5A7 as a template. 

The amplified vNAR fragments and the pSEX81 vector are digested with *Nco*I and *Not*I and ligated to construct the recombinant phagemid ((Figure 1A①). Then, the ligated phagemid is transformed into electrocompetent *E. coli TG1* and plated on 2X TY-GA agar plate (Figure 1A②–③). 90 clones of this produced library are sent for sequencing for qualification and quantification of the library. Afterwards, collected clones are used for infection with helper phage and production of phage antibody library (Figure 1B④). After infection, amplified phages that are present in the supernatant are precipitated and ready for selection of binders (Figure 1B⑤). Panning is performed according to Hust et al. [23] with some improvements. Here, four panning rounds against recombinant human tumor necrosis factor alpha (TNF alpha) are described. But, this protocol can be used for any other protein antigen, as well. When the antigen is incubated with the phages, the wells are washed stringently, and the phages are eluted by incubation with trypsin for proteolytic cleavage. The eluted phages are then used to infect *E. coli TG1* bacteria and titers are determined by plating of different dilutions. For subsequent rounds of selections, colonies from the first round are scraped from agar plates, and phages are produced in liquid culture, PEG-purified and selected by binding to antigen. After three or four rounds of panning, 10^9^ phages of each round are used for a polyclonal phage ELISA to evaluate the enrichment (Figure 1C⑥). Individual clones are isolated from the enriched rounds of panning (usually last round of panning), grown overnight, and soluble fragments are produced in a 96-well plate format (Figure 1C⑦). ELISA identifies antigen-specific monoclonal binders (Figure 1C⑧). Finally, the positive clones are sequenced and analyzed. Based on the ELISA results, suitable clones can be selected and sub-cloned into either a prokaryotic or a eukaryotic expression plasmid for protein production.

### 2.1. Materials

- MICROLON^®^ microplate, 96-well, F-bottom, high-binding (Greiner, Frickenhausen, Germany; Cat. no.: 655 061)
- Cellstar^®^, 96 Well Suspension Culture Plate, U-bottom (Greiner, Frickenhausen, Germany; Cat. no.:650001)
- PCR grade water (Thermo Fisher Scientific, Darmstadt, Germany; Cat. no.: AM9932)
- dNTP mix (Thermo Fisher Scientific, Darmstadt, Germany; Cat. no.: R0191)
- Platinum™ II Hot-Start Green PCR Master Mix (2X) (Thermo Fisher Scientific, Darmstadt, Germany; Cat. no.: 1400101)
- T4 DNA ligase (5 U/µL; Thermo Fisher Scientific, Darmstadt, Germany; Cat. no.: EL0011)
- Agarose (Carl Roth, Karlsruhe, Germany; Cat. no.: 11388983001)
- 100 base pair(s) (bp) ladder (New England Biolabs, Frankfurt am Main, Germany; Cat. no.: N0551G)
- *Nco*I Restriction Enzyme (New England Biolabs, Frankfurt am Main, Germany; Cat. no.: R0193)
- *Not*I Restriction Enzyme (New England Biolabs, Frankfurt am Main, Germany; Cat. no.: R0189)
- Anti-human IgG Peroxidase conjugate (Sigma-Aldrich Chemie GmbH, Munich, Germany; Cat. no.: A0545)
- Anti-M13 pIII monoclonal antibody (New England Biolabs, Frankfurt am Main, Germany; Cat. no.: E8033)
- Anti-M13 pVIII filamentous phages antibody (PROGEN, Heidelberg, Germany; Cat. no: 65197)
- Anti-mouse IgG Peroxidase conjugate (Sigma-Aldrich Chemie GmbH, Munich, Germany; Cat. no.: A8924)
- Anti-Penta-His ^TM^ monoclonal antibody (Merck Millipore, Darmstadt, Germany; Cat. no.: 70796)
- GeneRuler™ DNA Ladder Mix (Thermo Fisher Scientific, Darmstadt, Germany; Cat. no.: SM0331)
- Lysozyme (Carl Roth, Karlsruhe, Germany; Cat. no.: 8259.1)
- Maximo Taq DNA Polymerase (GeneOn, Ludwigshafen, Germany; Cat. no.: S101)
- Ampicillin (Sigma-Aldrich Chemie GmbH, Munich, Germany; Cat. no.: A9393)
- Kanamycin (Sigma-Aldrich Chemie GmbH, Munich, Germany; Cat. no.: 60615)
- Trypsin (Sigma-Aldrich Chemie GmbH, Munich, Germany; Cat. no.: T1426)
- *E. coli TG1*: F′ [traD36 proAB+ lacIq lacZΔM15] supE thi-1 Δ(lac-proAB) Δ(mcrB-hsdSM)5, (rK-mK-) (Lucigen, Middleton, USA; Cat. no.: 60502).
- Phagemid pSEX81 (PROGEN, Heidelberg, Germany; Cat. no: PR3005)
- Hyperphage (PROGEN, Heidelberg, Germany; Cat. no. PRHYPE-XS)
- Oligonucleotide primers for PCR are listed in Table 1.
- Gene Pulser^®^/MicroPulser™ Electroporation Cuvettes, 0.2 cm gap (Micropulser™, Bio-Rad, Munich, Germany; Cat. no.: 1652082)
- PEG-8000 (Sigma-Aldrich Chemie GmbH, Munich, Germany; Cat. no.: 729108-1G)
- Monarch™ DNA Gel Extraction Kit (New England Biolabs, Frankfurt am Main, Germany; Cat. no.: T1020G)
- M13KO7 helper phages (New England BioLabs, Frankfurt am Main, Germany; Cat. no.: N0315S)
- 1-Step™ Ultra 3,3′,5,5′-Tetramethylbenzidine-enzyme-linked immunosorbent assay (TMB-ELISA) (Thermo Fisher Scientific, Darmstadt, Germany; Cat. no.: 34028)
- 6X DNA Gel Loading Dye (Thermo Fisher Scientific, Darmstadt, Germany; Cat. no.: R0611)
- Agar-Agar Carl Roth (Carl Roth, Karlsruhe, Germany; Cat. no.: 6494.4)
- Agarose (Biozym Scientific, Oldendorf, Germany; Cat. no.: 840004)
- 4-(2-hydroxyethyl)-1-piperazineethanesulfonic acid (HEPES) (Carl Roth, Karlsruhe, Germany; Cat. no.: 9105.4)
- Roti-Safe Gel Stain (Carl Roth, Karlsruhe, Germany; Cat. no.: 3865.1)
- Tryptone/Peptone (Carl Roth, Karlsruhe, Germany; Cat. no.: 8952.4)
- Tween^®^ 20 (Carl Roth, Karlsruhe, Germany; Cat. no.: 9127.2)
- Yeast extract (Carl Roth, Karlsruhe, Germany; Cat. no.: 2363.1)
- Roti^®^-Prep Plasmid MINI (Carl Roth, Karlsruhe, Germany; Cat. no.: HP29.2)
- GeneJet PCR purification kit (Thermo Fisher Scientific, Darmstadt, Germany; Cat. no.: K0702)

### 2.2. Oligonucleotides

The oligonucleotides used in this protocol are listed in Table 1. The primers ForFR1_NcoI and RevFR4_NotI are designed to add the restriction enzyme sites *Not*I and *Nco*I to both ends of the vNAR gene. RevRan_13-20 primers contain NNK repeats used to randomize the CDR3 region of the vNAR during PCR. 

### 2.3. Equipment

- Toptable Centrifuge 5427R (Eppendorf, Hamburg, Germany; Cat. no.: 5409000012)
- Electrophoresis gel tank and power pack (VWR Peqlab, Lutterworth, UK; Cat. no.: 700-0444)
- Electroporator Gene Pulser^®^ (Micropulser™) (Bio-Rad, Munich, Germany; Cat. no.: 1652100)
- Fusion FX Gel Documentation (Vilber Lourmat, Collégien, France; Cat.: no.: 826)
- New Brunswick Innova 4200 Incubator (Eppendorf, Hamburg, Germany; Cat. no.: NB-4200)
- NanoDrop 1000 Spectrophotometer (Thermo Scientific, Waltham, MA, USA; Cat.: 25627)
- PCR Cycler Eppendorf Mastercycler (Eppendorf, Hamburg, Germany; Cat. no.: 5332)
- SpectraMax M Series Multi-Mode Microplate Readers (Molecular Devices, San Jose, CA, USA; Cat. no.: MLDVM2)

### 2.4. Software

- Vector NTI Advance 11
- GraphPad Prism version 7 Software (GraphPad Software Inc., La Jolla, CA, USA)
- CLC Main Workbench 8.0.1 (QIAGEN Redwood City, CA, USA)

## 3. Procedure

**Figure 2:**
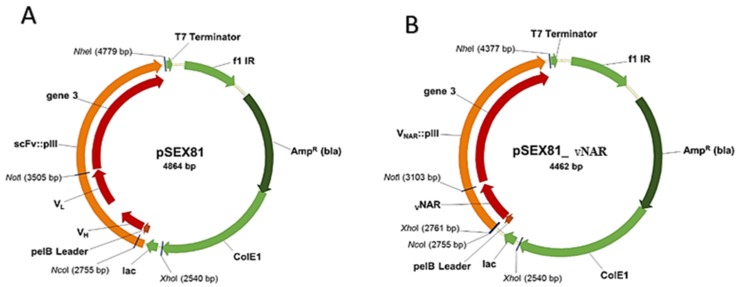
Vector map of phagemid pSEX81. (**A**) Original pSEX81 phagemid with single chain variable fragments. (**B**) Recombinant pSEX81_vNARs. The variable domain of shark antibody is cloned into a pSEX81 plasmid by *Nco*I and *Not*I restriction enzymes, in fusion with the pelB leader at the N-terminal and pIII at the C-terminal part of the antibody.

The phagemid pSEX81 is used for the construction of the library and preparation of phage antibody particles. This vector is designed for the expression of functional recombinant scFv-pIII fusion proteins on the surface of M13 bacteriophages. It further contains the bacterial pectate lysate (*pelB*) signal sequence responsible for periplasmic transport of the fusion proteins. It also codes for an ampicillin resistance by β-lactamase for antibiotic selection. The antibody-pIII fusion protein can be overexpressed by Isopropyl β-D-1-thiogalactopyranoside (IPTG) induction through the lactose β-galactosidase (LacZ) promotor. Finally, the vector contains the bacterial ColE1 ori and the intergenic region of phage f1 as the origin of replication. The plasmid map and the used restriction enzyme *NcoI* and *NotI* locations are depicted in Figure 2.

For the first round of panning, hyperphages are used to infect the library for oligovalent display. For the subsequent panning rounds, helper phages are used for monovalent display. This will increase the selection stringency.

Affinity maturation may be performed to improve the binding of a selected antibody by introducing more diversity into hypervariable regions 1 or 2 or CDR1.

### 3.1. In vitro Synthesis of Variable DNA Fragments and Cloning into the Phagemid pSEX81

Time for Completion: 3 days

1. For each reaction with different randomized reverse primer, add the followings material to nuclease-free water to make a final volume of 50 µL in a PCR tube on ice: 25 µL of Platinum™ II Hot-Start Green PCR Master Mix, 10 µL Platinum™ GC Enhancer (provided by PCR master mix), 5 ng of template DNA (HEL-5A7) and finally add 1 µL of each 10 µM primers (final concentration 0.2 µM). Use ForFR1_NcoI as forward primer and one of the randomized reverse primers (RevRan_12 to RevRan_23) for each reaction. Mix well and briefly centrifuge the contents.
2. Perform PCR with the following set up: initial denaturation at 94 °C for 2 min. Then proceed for 20 cycles as follows: 5 s at 98 °C, annealing at 60 °C for 15 s and elongation of fragments for 15 s at 68 °C.
3. Load PCR samples onto a 1% agarose gel and run the gel at 110 V. Inspect the gel under UV light. Excise the bands around 350 bp length and extract them from the agarose gel using the DNA gel extraction kit, according to manufacturer’s protocol. Elute the DNA of each PCR reaction with 10 µL nuclease-free water.
4. Use the eluted PCR product for a second PCR to introduce restriction digestion sites. Proceed as it is described in step 2, use ForFR1_NcoI and RevFR4_NotI as primers for the reaction.
5. Digest both, the PCR products and the pSEX81 plasmid with *Nco*I and *Not*I. For this, prepare a total volume of 50 µL digestion reaction for the PCR products (at least 4 µg) by adding 2 µL (20 units) of each enzyme *Nco*I and *Not*I, 5 µL of 10X NEB3 buffer and adjust the volume reaction up to 50 µL with nuclease-free water. For the plasmid, digest 20 µg of the pSEX81 vector, add 15 µL of 10X NEB3 buffer, 10 µL (100 units) of each enzyme *Nco*I and *Not*I and add nuclease-free water up to 150 µL. Incubate for 3 h at 37 °C following 10 min at 65 °C.
6. After restriction digestion, purify DNA fragments using GeneJet PCR purification kit according to the manufacturer’s instruction. Elute each purified digested sample in 20 µL water.
7. Run the digested plasmid on a 1% agarose gel and cut out the target band quickly with a scalpel on a UV-transilluminator table. Purify the DNA fragments from the agarose gel using the Monarch™ DNA Gel Extraction Kit, following the manufacturer’s instructions. Elute in 100 µL preheated sterile H_2_O. Determine the concentration by Nanodrop 1000 spectrophotometer.
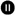
**PAUSE STEP**: The extracted and purified digested DNA fragments can be stored at −20 °C until use.
8. Ligate the digested fragments by preparing following material: Calculate the amount of needed insert for 100 ng of the plasmid with these molar ratios: 1:0.5, 1:1, 1:2 vector: insert. Add 1 µL of ligase, 1 µL of Ligase buffer (10X) and water to 10 µL. Keep at 16 °C overnight following 10 min at 65 °C.
9. Transform 2 µL of ligation reaction by electroporation into TG1 cells. Plate 100 µL of 10^−2^, 10^−3^, and 10^−4^ dilutions on 2X TY-GA agar plates and incubate overnight at 37 °C.
10. Measure the cloning efficiency by colony PCR with For_pSEX and Rev_pSEX primers. For this, pick 50 individual clones, mix each clone with 20 µL of water and cook them at 95 °C for 10 min. Later centrifuge them and take 2 µL of the supernatant as a template for the PCR.
11. Based on the result of cloning efficiency, prepare the final ligation reaction for the best-determined ratio. For each insert with specific CDR3 length, add 2 µg of plasmid and an appropriate amount of each insert into 100 µL reaction volume. Incubate at 16 °C for 16 h, heat for 10 min at 65 °C and purify using GeneJet PCR purification kit. Elute each reaction in 20 µL and pool all the sample together.
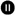
**PAUSE STEP:** After stopping the reaction, the mix can be stored at −20° C until further use.

### 3.2. Transformation in Electrocompetent TG1 and Library Construction. 

Time for Completion: two days or four days including preparation of competent cells

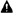
**CRITICAL STEP:** In order to increase the transformation efficiency, prepare electrocompetent cells on the same day of transformation and avoid freezing the cells before transformation.

#### 3.2.1. Preparation of Electrocompetent TG1. 

The protocol described here is based on the method from the Sidhu and co-workers [2,24].

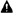
**CRITICAL STEP:** Chill all the material including HEPES, glycerol, H_2_O, pipettes and tips at 0 °C. Centrifuge and rotor must be precooled to 2 °C.

12. Pick a fresh clone of *E. coli* TG1 from 2X TY-agar plate into 20 mL 2X TY medium in a 125 mL flask and incubate at 37 °C with vigorous shaking at 220 rounds per min (rpm) overnight.
13. The next day, add the overnight culture to 1 L fresh 2X TY in a 5 L flask (with starting OD_600_ at around 0.1) and incubate at 37 °C and 180 rpm until an optical density OD_600_ of 0.7.
14. Distribute the flask content equally into two sterile 500 mL centrifuge bottles and cool down on ice for 30 min.
15. Centrifuge for 5 min with 5000× *g* at 2 °C. All subsequent centrifugation steps should be done with 5000× *g* at 2 °C.
16. Remove the supernatant of both bottles carefully and re-suspend each pellet in 500 mL cold H_2_O/HEPES (see reagent setup). Add a sterile magnetic stir bar to each bottle to completely dissolve the pellets on a magnetic stirrer.
17. Centrifuge for 10 min and repeat the previous step with another 500 mL cold H_2_O/HEPES.
18. Discard the supernatant and resuspend each pellet in 50 mL cold glycerol/HEPES. Pool the bacteria suspension of both bottles into a fresh sterile centrifugation bottle without transferring the stir bars.
19. Centrifuge for 15 min. Remove the supernatant and re-suspend the pellet in 1 mL cold glycerol/HEPES (see reagent setup).
20. Distribute this volume into 300 µL aliquots. Use immediately for transformation.

#### 3.2.2. Transformation of Electrocompetent *E. coli* TG1 by Electroporation

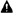
**CRITICAL STEP:** Cool at least 6 cuvettes and slides of the gene pulser down to 2 °C. For each control and test sample, prepare 0.950 mL of SOC in 2 mL tubes and 5 sterile 100 mL Erlenmeyer flasks with 25 mL SOC each for the samples.

21. Apply this setting for the gene pulser: 2.5 kV and 25 µF.
22. For negative control, dry the aluminum electrodes of the first cuvette with tissue paper, add 50 µL of electrocompetent cells to the cuvette, put the cuvette in the holder, push in into the gene pulser and start the electric pulse. Now, the cells are very fragile and you need to add pre-warmed SOC medium immediately. Rinse the cuvette a few times with SOC medium and put it quickly into the incubator.
23. For the positive control add 1 µL of highly pure pSEX81 plasmid to 50 µL of the cells and perform the electroporation as described above.
24. Add 10–15 µL of purified ligation mixture to 300 µL of electrocompetent *E. coli*. Gently tab the bottom of cuvette on the bench to avoid bubble formation. After electroporation, immediately transfer the cells into the 125 mL flask and wash the cuvette with the prewarmed medium.
25. Repeat step 24 for the other ligation reactions.
26. Shake the entire samples at 37 °C in 220 rpm for 45–60 min.
27. Pool the library and plate 100 µL of 10^−2^ to 10^−5^ dilutions of the library, 10^−1^ and 10^−2^ dilutions of the positive control and 100 µL of the undiluted negative control on 2X TY-GA plates.
28. Centrifuge the rest of library suspension at 3220× *g* for 10 min and discard the supernatant. Resuspend the pellet in 5 mL SOC medium and plate on five 150-mm 2X TY-GA plates.
29. Incubate the plates overnight at 37 °C.
30. To estimate the transformation titer, plate 100 μL of 10^−3^ and 10^−4^ dilutions of the cells on 2X TY-GA agar plates.
31. The transformation titer is calculated as:
  $$\frac{no.\;of\;colonies\times \left(ml\;recovery\;medium\;culture\right)\times 1000\;\left(\mu l/ml\right)}{100\;\left(\mu l\;plated\right)\times dilution\;fold\;\left(10^{-3}\;or\;10^{-4}\right)}$$
32. Centrifuge the remaining cells (2000× *g*, 15 min), and resuspend the pellet in 1000 μL of 2X TY medium. Plate the suspended bacteria on a 150 mm diameter 2X TY-GA agar plate and incubate overnight at 37 °C.
33. The next morning, add 5 mL of SB medium to the 150 mm diameter agar plates and scrape the bacteria using flame-sterilized glass spreader. Add glycerol to the final concentration of 10% glycerol and mix well.
34. Prepare 1 mL aliquots, and store at −80 °C.

### 3.3. Phage Preparation and Selection of vNARs against Antigen by Phage Display

Total time for completion: 3–4 weeks (includes 3 days for packaging, 2 weeks for panning, 2 days for packaging, 2 days for phage titration)

#### 3.3.1. Packaging of Synthetic Library Employing Hyperphage (M13KO7ΔpIII).

35. Thaw aliquot of frozen antibody library on ice. Add it to 500 mL 2X TY-GA medium. The initial OD_600_ should be approximately 0.1. Grow the culture at 37 °C, 250 rpm in a 2 L glass flask until OD600 = 0.5 (approximately 1.5–2 h).
36. Add 1 × 10^12^ M13KO7 hyperphages to the culture and incubate at 37 °C without shaking for 15 min following 30 min vigorous shaking.
37. Spin the culture at 3200× *g* for 15 min and discard the supernatant.
38. Resuspend pellets in 500 mL of 2X TY-AK medium.
39. Grow for 16–20 h at 28 °C, 250 rpm in a 2 L glass flask.
40. The next day, centrifuge the culture for 20 min at 5500× *g* and 4 °C.
41. Add 1/4 volume of PEG/NaCl solution (see Reagent setup) to precipitate the produced phage binder from the culture supernatant.
42. Following four hours of incubation on ice, pellet the antibody phages by centrifugation for 60 min at 5500× *g* and 4 °C.
43. Re-suspend the white phage pellet in 1 mL PBS.
44. To remove *E. coli* particles and bacterial debris, centrifuge the solution three times for 5 min.
45. Collect the supernatant and transfer it to a new 1.5 mL tube containing 250 µL of PEG/NaCl.
46. Incubate it on ice for 30 min. Then pellet the precipitated phages by centrifugation at 17,900× *g* for 10 min and discard the supernatant.
47. To remove the final traces of PEG, centrifuge again for another 2 min at 17,900× *g* and carefully remove the remaining supernatant.
48. Re-suspend the phages in 0.5 mL PBS. This supernatant contains the phage particles. Store them at 4 °C. These phages are used for the first round of panning.

#### 3.3.2. Panning of vNAR Library against an Antigen (Here TNF Alpha Was Used)

49. For the first panning round, use 4 µg protein/well per panning, for the following rounds use 1 µg protein/well for more stringent conditions. Dissolve the antigen and incubate in a microtiter plate well overnight at 4 °C. Coating conditions (incubation temperature and coating buffer) should be set-up for the antigen of interest. Prepare overnight culture of TG1 cells grown on minimal medium (M9) by transferring 1 colony from fresh culture to 5 mL LB. Growth of TG1 on minimal medium selects for cells with F’ factor.
50. For biopanning of the synthetic library, coat 2 µg of the antigen TNF alpha in 150 µL of 50 mM NaHCO_3_ (pH 9.6) on a 96-well well plate incubated at 4 °C overnight
51. The following day, remove the coating solution from the plate by tapping on a clean tissue. Wash the coated well three times with PBST in an ELISA washer or squirt bottle and block with 300 µL MPBST for 2 h at room temperature (RT).
52. After blocking, wash the well once with PBST and one to three times with PBS.
53. For binding of the antibody phages, incubate 10^11^ antibody phages in 200 µL blocking buffer for 1 h without shaking and 1 h at RT and gentle agitation (200 rpm). Meanwhile, inoculate 5 mL 2X TY with 500 µL of the overnight culture of *E. coli* TG1 and grow at 37 °C, 250 rpm to an OD_600_ 0.5. The culture can be kept at 4 °C until use. For the following round of panning change the blocking buffer to BSA-PBST to eliminate nonspecific binding of phages to the blocking buffer.
54. Discard the unbound phages by washing 10 times with PBST and 10 times with PBS for 2 min each. Increase the Tween^®^ 20 concentration by 0.1% for each washing step. For the subsequent panning rounds, increase the washing steps to 15 (round 2) and 20 (round 3 and 4) steps with PBST, respectively.
55. Elute phage particles by using 50 µL trypsin solution (corresponding to Solution A in Table 2) for 30 min at 37 °C. Repeat this step two times. Decrease the incubation time to 20 min. This will ensure the detachment of all bound phage particle and increase the yield of the panning process. Remove the supernatant containing the eluted phages and store it at 4 °C for the infection.
56. Add 100 µL of a log-phased *E. coli* TG1 to the trypsinated well and infect 500 µL of a log-phased *E. coli* TG1 culture in 2X TY medium with the eluted phages from step 55. Incubate both, first for 30 min at 37 °C without shaking and then the following 30 min with moderate shaking (250 rpm). Combine both suspensions.
57. After infection, plate 100 µL of the total volume from step 56 in serial dilutions (10^−2^ to 10^−4^ in 2X TY medium) on 2X TY-GA agar plates and incubated overnight at 37 °C.
58. Centrifuge the rest of the suspension for 20 min at 3200× *g* at RT.
59. Suspend the pellet in 1000 µL 2X TY-GA medium and incubate overnight at 30 °C and 200 rpm.
60. The next day, centrifuge the suspension for 10 min at 3200× *g* and suspend the pellet in 1000 µL fresh 2X TY-GA medium.
61. Scrap the colonies from the agar plates and add to the suspension. Add 100 µL glycerol (100%) and mix thoroughly.
62. Prepare 100 µL aliquots and store at –20 °C until further use.

#### 3.3.3. Packaging of Phagemid

63. For the subsequent round of panning, the eluted phages should be packaged and reamplified. Inoculate 1 mL of 2X TY-GA medium with 20 µL of the glycerol stock obtained after panning. Grow the culture for about 3 h at 37 °C 200 rpm.
64. Infect the cells with 2 × 10^9^ cfu (colony forming units) of M13KO7 helper phages and incubate for 1 h at 37 °C and 200 rpm.
65. To remove the medium, centrifuge the infected culture for 30 min at 3200× *g* and 4 °C. Discard the supernatant.
66. Suspend the pellet in 500 µL 2X TY/AK medium and incubate for 16 h at 30 °C 200 rpm for phage production.
67. The next day, pellet the bacteria twice by centrifugation for 10 min at 3200 rpm and 4 °C to precipitate the phages. Add 1/5 volume of PEG/NaCl solution to the supernatant and incubate for 1 h on ice.
68. To pellet the phages, centrifuge the solution for 1 h at 3200× *g* and 4 °C.
69. Resuspend the white phage pellet in 100 µL PBS and store at 4 °C.

#### 3.3.4. Phage Titration.

70. Make serial dilutions of the phage suspension in PBS. The number of eluted phage depends on several parameters (e.g., antigen, library, panning rounds, washing stringency).
71. Infect 50 µL bacteria with 10 µL of different phage dilutions (10^−6^, 10^−7^, 10^−8^) and incubate 30 min at 37 °C.

- Note: Check all solutions for phage contamination. To check the PBS or PEG solutions, use 10 µL of these solutions for *E. coli* “infection”. In parallel, plate out non-infected TG1 to check the bacteria.

72. Plate the 60 µL infected bacteria on 2X TY-GA agar plates.
73. Incubate the plates overnight at 37 °C.
74. Count the colonies and calculate the cfu or cfu/mL titer according to the dilution:
  $$\frac{cfu}{µl}=\frac{number\;of\;colonies}{\left(the\;volume\;of\;phages\;used\right)\;x\;\left(dilution\right)}$$

### 3.4. Screening and Selection of Antigen Binders. 

Total time for completion (without sequencing): 3–4 days 

(includes 5–6 h for polyclonal ELISA, 1–2 days for production, 7–8 h for monoclonal ELISA).

#### 3.4.1. Polyclonal Phage ELISA

The enrichment of antigen-binding phages is evaluated via a polyclonal phage ELISA. Here, the vNARs from each round of panning are displayed on the tip of the phages. Therefore, the immobilized phages are detected by an antibody against pVIII expressed on the phages’ surface.

75. For this, coat a 96-well plate with 50 ng of TNF alpha in 100 µL 50 mM NaHCO_3_ (pH 9.6) in duplicate for each round of panning and incubated overnight at 4 °C.
76. The following day, wash the coated wells three times with 200 µL PBST and then block with 300 µL MPBST for 2 h at RT.
77. After three washing steps with each 200 µL PBST, add 10^9^ cfu phage amplificate from each panning round to each well and incubate the plate for 1.5 h at RT.
78. After incubation, wash the wells three times with 200 µL PBST
79. Add 100 µL of a 1:100 dilution of anti-pVIII monoclonal antibody in blocking buffer and incubate for an additional 1 h at RT.
80. Wash the plates as above, and add 100 µL of a 1:10,000 dilution of the secondary antibody anti-mouse IgG peroxidase conjugate in blocking buffer and incubate for a further hour at RT.
81. After three washing steps add 100 µL of 1-Step™ Ultra TMB-ELISA substrate for color development to each well.
82. Stop the reaction by the addition of 100 µL of 1 M sulfuric acid (H_2_SO_4_).
83. Measure the absorbance at 450 nm using a SpectraMax Reader or a similar absorption reader.

#### 3.4.2. Production of Soluble Monoclonal Antibody Fragments in Microtitre Plates

The soluble vNARs are produced in fusion with pIII of the phagemid (vNAR-pIII, Figure 1C⑧). Therefore, the soluble fragments are detected with an anti-pIII antibody in the ELISA described below.

84. Take 20 µL of phage-antibody particles either from the last round of panning or the one with the highest enrichment, add 50 µL of trypsin solution and incubate for 30 min at 37 °C.
85. Infect 500 µL exponentially growing TG1 bacteria for 30 min at 37 °C.
86. Prepare different dilution series; 10^−2^–10^−4^ and plate them on 2X TY-GA and incubate overnight at 37 °C.
87. The next day, fill each well of a 96 well U-bottom polypropylene plate with a total volume of 1 mL per well with 100 µL 2X TY-GA.
88. Inoculate each well with an individual clone using sterile tips. Consider two wells for negative control to check the contamination of the wells and two wells for positive clone (containing pSEX81-Hel5A7). Seal the plate with a breathable sealing film.
89. Incubate overnight at 37 °C and 250 rpm.
90. Transfer 10 µL of overnight culture to a new 96 well plate and add 150 µL 2X TY-GA. Incubate at 37 °C and 250 rpm for approximately 2–3 h to reach an OD_600_ of 0.7–0.9. To the rest of the overnight culture add 10 µL glycerol, mix well, and store at −80 °C.
91. Centrifuge the plate for 10 min at 4000 rpm, remove the medium carefully and add 150 µL 2X TY medium, suspend the pellet and centrifuge again.
92. Add 150 µL 2X TY-A with 100 µM IPTG and incubate overnight at 30 °C and 250 rpm.
93. Centrifuge for 10 min at 4000 rpm. Transfer the antibody fragment containing supernatant to a new polypropylene plate (first supernatant) and store at 4 °C.
94. Add 100 µL of the periplasmic buffer to the pellet, resuspend and incubate on ice for 30 min.
  Centrifuge the plate, remove the supernatant (second supernatant) and combine it with the first supernatant. Keep at 4 °C until the next step.

#### 3.4.3. ELISA for Detection of Antigen Binding Monoclonal Soluble Antibody Fragments

95. Coat 50 ng of antigen into 96 well plates in 100 µL of carbonate buffer (50 mM NaHCO_3_; pH 9.6) or PBS for coating. For the positive control, coat 100 µL of 1 mg/mL of lysozyme and 50 ng of BSA as a negative control. Incubate overnight at 4 °C or 1 h at 37 °C.
96. Next day, wash the coated wells three times with 200 µL PBST and then block with 300 µL MPBST for at least 1 h at RT.
97. Mix 100 µL of soluble antibody fragments (combined first and second supernatant) with 100 µL of blocking buffer and add them to coated wells. Incubate for 90 min at RT.
98. Wash 3 times with PBST.
99. As a primary antibody, use a 1:1000 dilution of an anti pIII monoclonal antibody and incubate for 1 h at RT.
100. Add secondary antibody and proceed with the steps 80–83 of Section 3.4.1.

#### 3.4.4. Sequencing

101. To determine the nucleotide sequence of plasmid DNA, DNA samples should be sent to sequencing. The sequencing of our sample was performed at GATC BIOTECH AG (Köln, Germany). The resulting data of the sequencing can be analyzed using the software of Vector NTI or CLC Main Workbench 8 or any similar software.

## 4. Expected Results

### 4.1. In Vitro Synthesis of Variable DNA Fragments

**Figure 3:**
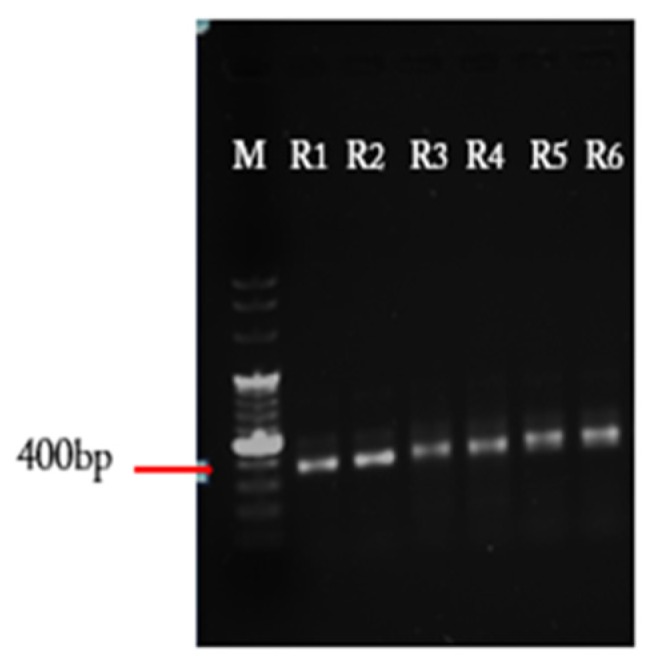
Amplified vNAR fragments with randomized CDR3 separated on 1% agarose gel. M: 6 µL GeneRuler™ DNA Ladder Mix is used as a marker. R1 to R6: PCR reactions with degenerated CDR3 primers with the length of 12, 13, 14, 18, 20 and 23 bp, respectively.

After the amplification of DNA fragment using the reverse randomized primer with different lengths, you will get different bands from 350 to 400 bp based on each reverse primer used for the PCR reaction (see Figure 3). The PCR reaction can be improved by decreasing the template and/or primers.

### 4.2. Colony PCR

**Figure 4:**
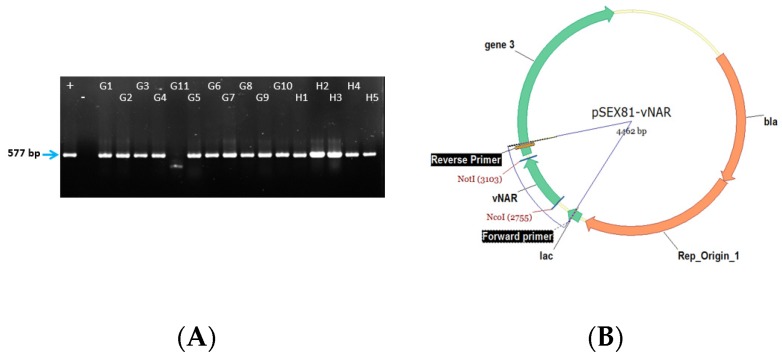
(**A**) Gel electrophoresis of colony-PCR from randomly picked clones to estimate the correct insertion rate. Positive clones are defined by having an insert in the range 570–600 bp (+, positive control HEL-5A7; −, negative control H_2_O). (**B**) The position of primers used for colony PCR is shown on the plasmid map (bla, beta-lactamase/ampicillin resistance).

Colony PCR is used to evaluate the quality of cloning and transformation and to determine whether the insert is present in the plasmid. The position of primers on the plasmid and expected size of the fragments are shown in Figure 4B. For a successful construction of the antibody library, at least 90% of the clones should give a PCR band (Figure 4A). This will ensure that it can be used for the selection of binders against the antigen of interest.

### 4.3. Sequencing of Selected Clones from vNAR Library with Randomized CDR3

**Figure 5:**
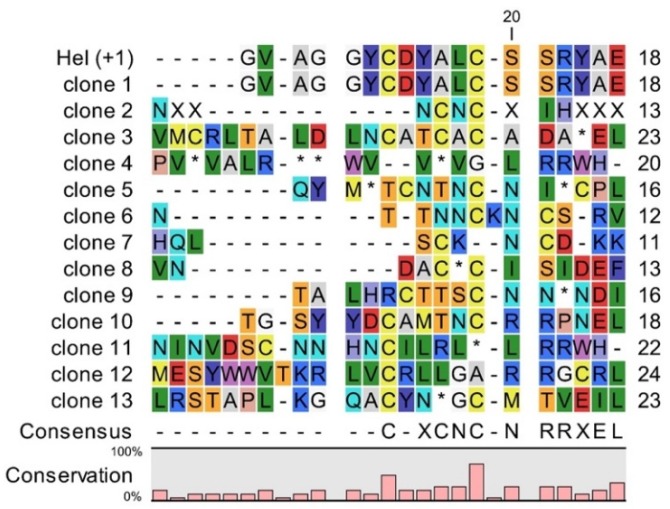
The amino acid composition of a synthetic vNAR library with variable CDR3: Representative sequences of different clones are compared to the template sequence (Hel-5A7). For simplicity, only CDR3 is presented here. Stop codons are shown as an asterisk (*) and a dash (-) represents deletion of an amino acid.

To further evaluate the quality of the library, the distribution of amino acids in the artificial CDR3 libraries needs to be assessed via sequencing. As shown in Figure 5, the clones of the library constructed with a randomized primer for CDR3 differed in the length and composition of their CDR3. Figure 5 shows some possible sequences for CDR3 when using randomized primers. Some clones will be the same as the used template. For this synthetic library, 5% of the sequenced clones were identical to the template Hel-5A7. To decrease the number of identical clones, it is recommended to either use as less template as possible for the PCR reaction or to digest the parental DNA with *Dpn*I in case the parental DNA plasmid was propagated in standard *E. coli* strains. Also, frameshift mutations can be detected in amplified fragments, although the primers used contain only multiples of 3 codons. These errors introduced by oligonucleotides can lead to nonsense codon (clone 2) or very different sequence in CDR3 and the rest of vNAR framework 4 (clone 3). Such framework shifts occurred in 40% of the sequenced clones. Errors in primers also can lead to deletion of conserved cysteine, which prevents the formation of the stabilizing disulphide bond (clone 4). Presence of amber stop codon (clone 5) occurred in 8% of the sequenced clones. As long as *SupE* strains of *E. coli* are used in phage display, this codon can be mostly read as glutamine (Q). Finally, other clones will represent the array used in primers with different length and two conserved cysteines (clone 6 to clone 13). By using this protocol and by ensuring that the library is large enough, the number of clones with the above-mentioned problems is rather low, and a sufficient amount of binding protein against the antigen of interest can be obtained.

### 4.4. Enrichment of Panning Round

**Figure 6:**
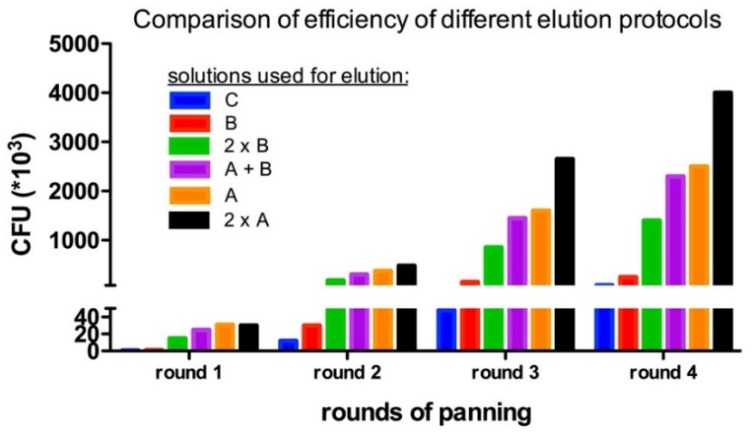
The number of transforming phages after each round of panning varies with different elution methods. Two subsequent trypsinization steps in solution A clearly demonstrated the highest elution efficiency of infectious phages.

One critical step in each panning round is the elution of bound phages with their displayed vNAR to the antigen of interest. So, harsh washing steps are used in this process. For example, the number of washing steps is increased and the concentration of Tween^®^ 20 is also increased in subsequent panning round. In addition, non-specific elution may cause a problem. Rather harsh conditions are needed to elute strong binders, but they shall not harm the quality of the phages. The titer of the eluted phages is determined to evaluate the enrichment and thereby the quality of this panning step after each round of panning. After using standard conditions for elution (i.e., solution C), a polyclonal ELISA was performed on the plate used for biopanning. Surprisingly, a high ELISA signal (data not shown) corresponding to a high number of phages that are still bound either specifically to the antigen or un-specifically to the well. Therefore, we tested several different methods to elute the phages after biopanning (Table 2 and Figure 6). Figure 6 shows the recovered phages after each panning around with different elution buffers. To sum up the results, using two trypsinization steps in solution A increased the number of colony forming units (cfu) that arose after infection with the eluted phages on average by 50-fold. Moreover, by adding the bacteria directly to the well with potentially remaining target-bound phages after the trypsinization, even more phages harboring potential good binders can be recovered by infecting the bacteria with their phagemid. In the above-presented protocol, all these steps are already included to guarantee an optimal result after biopanning.

**Figure 7:**
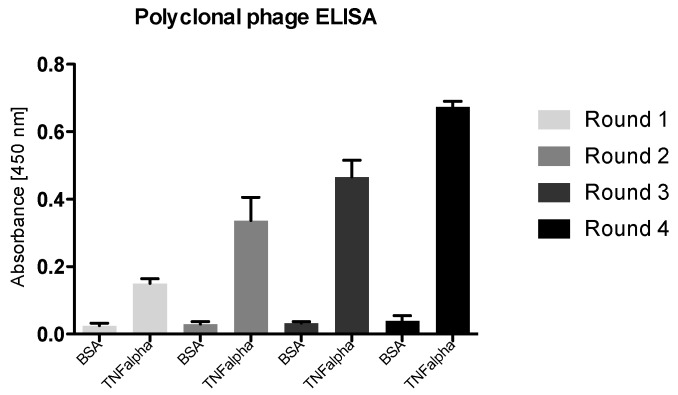
Polyclonal phage ELISA for all rounds of panning against TNF alpha as an antigen and BSA as a control. Phage pools of each panning round are exposed to immobilized TNF alpha. BSA was used as a control antigen for each panning round since BSA was used for blocking the wells. Increase in absorbance was observed during the selection rounds.

To check the enrichment of specifically binding phages, a polyclonal phage ELISA was performed. This ELISA provides essential information about the enrichment during the screening process in phage display. Here, a representative synthetic vNAR library was panned against the immobilized antigen TNF alpha and four rounds of biopanning were performed. The stringency of washing was increased each selection round while the amount of immobilized TNF alpha is decreased from one round to the next. The results of a representative polyclonal phage ELISA are shown in Figure 7: a steady increase in the antigen binding was observed for the polyclonal phage over the four selection rounds.

### 4.5. Monoclonal Selection of Antibody Fragments

**Figure 8:**
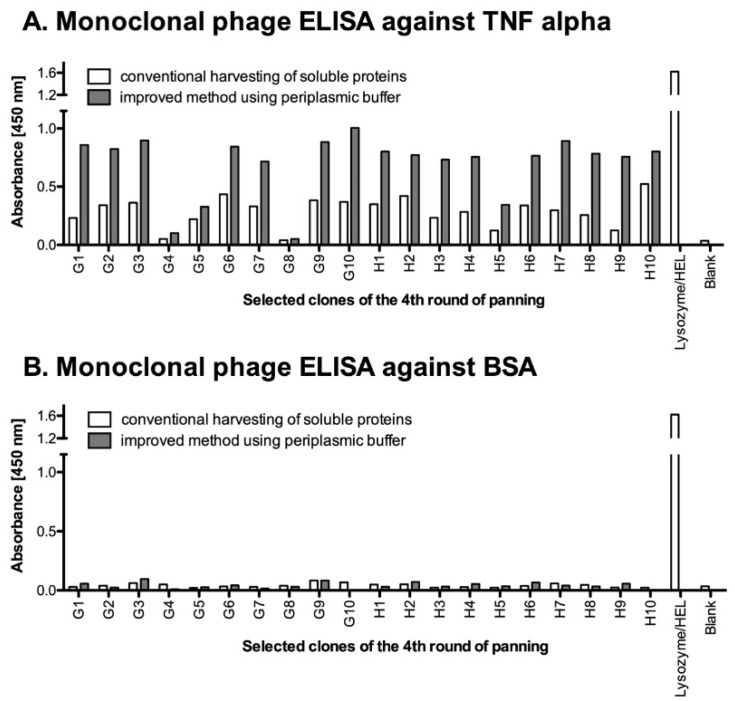
Enzyme-linked immunosorbent assay (ELISA) of soluble vNAR-pIII fusion proteins against (**A**) Tumor necrosis factor (TNF) alpha as antigen and (**B**) Bovine serum albumin (BSA) as control from selected clones of the most enriched round of panning. Data obtained with the conventional method are displayed as white bars and data from the improved method as grey bars. For higher throughput, clones were determined as n=1. HEL-5A7 was determined against coated lysozyme as a positive control and against BSA as blank.

After performing the panning rounds for selection, large numbers of enriched clones should be evaluated by polyclonal phage ELISA or by ELISA for the monoclonal soluble antibody fragments. Here, we describe a direct approach to analyze the selected clones based on their expression as functional vNAR–pIII fusion proteins and detection via an anti-pIII monoclonal antibody. The procedure for the production of soluble antibody fragments in fusion with pIII was adapted from Mersmann et al. [25] and Hust et al. [20]. Based on their methods for soluble expression in a 96-well format, these fusion proteins are produced and released into the medium by *E. coli* and can be harvested from the supernatants. But, we found out that in many cases the produced levels of soluble native proteins are too low for further characterization. Hence, a special periplasmic buffer was used to improve the recovery of proteins from the periplasmic space and to increase the concentration of vNARs in the supernatant. As shown in Figure 8, this procedure using both, the periplasmic and the supernatant fractions, improved the detection of the soluble fragments by two- to threefold compared to the conventional harvesting of the supernatant only. Clones with OD_450_ ten-fold higher to blank are chosen for further investigation. Figure 8 shows some representative clones selected from the 4th round of panning, which showed the highest binding to their antigen according to results of the polyclonal ELISA (see Section 4.4). Here, as a positive control, lysozyme was coated and detected with Hel-5A7, the starting template of this synthetic library. Hel-5A7 was cloned into phagemid vector and expressed under the same condition as other clones obtained from the 4th round of panning (see Section 3.4.2).

## 5. Reagents Setup

1. Kanamycin solution: Dissolve kanamycin powder at 50 mg/mL in deionized water. Filterthrough 0.2 µm filter. Aliquot in 1 mL portions. Can be stored at −20 °C.
2. Ampicillin solution: Dissolve ampicillin powder at 100 mg/mL in deionized water. Filterthrough 0.2 µm filter. Aliquot in 1 mL portions. Can be stored at −20 °C indefinitely. ThawedAliquots should be freshly diluted 1000-fold into medium or agar.
3. Glucose solution (40%): Dissolve 400 g of glucose in 1 L of deionized water. Filter through0.2 µm filter. Can be stored at 4 °C for several months.
4. 2X TY medium pH 7.0: 1.6% (w/v) tryptone, 1% (w/v) yeast extract, 0.5% (w/v) NaCl)
5. 2X TY-GA: 2X TY, 100 mg/mL ampicillin, 100 mM glucose
6. 2X TY-GA agar plates: Dissolve 10 g tryptone, 5 g yeast extract, and 10 g NaCl in 1L water. Add 15 g agar-agar and autoclave. Cool down below 50 °C, add 1 mL of filter-sterilized ampicillin (100 mg/mL) and 50 mL of 40% (w/v) filter sterilized glucose. Mix with gentle stirring and fill one-third of 100 mm or 150 mm diameter polystyrene Petri dishes. Keep the plates at 4 °C.
7. SB medium pH 7.0: Dissolve 30 g tryptone, 20 g yeast extract and 10 g MOPS (3-[N-morpholino]–propanesulfic acid) in 1L water. Dissolve and autoclave.
8. Minimal medium agar plate: Add 5.6 g 5X M9 salt and 15 g agar to 500 mL of deionized water and autoclave. When cooled to 50-45° C, add 1 mL of 1 M MgSO_4_ (autoclaved), 0.1 mL of 1 M CaSO_4_ (autoclaved), 5 mL of 40% glucose, and 0.25 mL of 1% thiamine HCl (filter-sterilized). Mix with gentle stirring and pour on polystyrene Petri dishes. Keep the plates at 4 °C.
9. SOC medium: Add 20 g of tryptone, 5 g of yeast extract and 0.5 g of NaCl to 950 mL of deionized water. Shake well and add 10mL of a 250 mM KCl. Adjust the pH of the medium to 7.0. autoclave for 20 min. Add 5 mL of a sterile solution of 2 M MgCl_2_.
10. Agarose electrophoresis gel: For 1% agarose gel, use 1 g of agarose and 100 mL TAE buffer (40 mM Tris, 20 mM acetic acid, and 1 mM EDTA, pH 8.0). Change the amount of agarose as needed to make 1.5 or 2% gels.
11. H_2_O/HEPES: Add 1 mL of sterile HEPES 1M to 1 L of autoclaved ultra-pure water.
12. Glycerol/HEPES: Add 1 mL of sterile HEPES 1M to 1L 10% glycerol.
13. TBSC buffer: 10 mM Tris pH 7.4, 137 mM NaCl, 1 mM CaCl_2_. Dissolve 1.5 g of TrisBase, 8 g NaCl and 0.15 g CaCl_2_ in 1 L of deionized water. Adjust to pH 7.4 and autoclave.
14. PEG solution: 20% PEG, 2.5 M NaCl. Dissolve 100 g of PEG 8000 and 73 g of NaCl in 500 mL of deionized water. Filter through 0.2 µm filter.
15. Trypsin solution: Dissolve trypsin powder at 10 mg/mL in TBSC (trypsin stock). Freeze in 20 µL aliquots in liquid nitrogen. This can be stored at −20 °C for several months. For the experiment, dissolve 100 µlL of trypsin stock in 10 mL of TBSC (trypsin solution).
16. PBST: 1X PBS + 0.1% (v/v) Tween^®^ 20 up to 1X PBS + 1% (v/v) Tween^®^ 20.
17. MPBST: 2% skimmed milk in PBST, prepare freshly.
18. Panning block solution: MPBST or BSA-PBST: 1% (w/v) BSA in PBST, prepare freshly.
19. Periplasmic buffer: 100 mM Tris; 0.5 M Sucrose; 1 mM EDTA; pH 8.0

## Figures and Tables

**Table 1:** List of used oligonucleotides. N: any base; A: Adenine; G: Guanine; C: Cytosine; T: Thymine; M means a 50% mix each of amino (A or C) nucleotides in reverse primers.

Name	Sequence (5′-3′)
ForFR1_NcoI	GCATGACCATGGCTCGAGTGGACCAAACACCG
RevFR4_NotI	GCATGAGCGGCCGCTTTATTCACAGTCACGGCAGTGCCAT
For_pSex	GTATGTTGTGTGGAATTGTG
Rev_pSex	GTTTTGTCGTCTTTCCAG
RevRan_12	CGGCAGTGCCATCTCCGCA(MNN)_3_GCA(MNN)_2_-ACA(MNN)_5_GAGACCGCAACGATACGTGCCAC
RevRan_13	CGGCAGTGCCATCTCCGCA(MNN)_6_GCA(MNN)_1_- ACA(MNN)_4_GAGACCGCAACGATACGTGCCAC
RevRan_16	CGGCAGTGCCATCTCCGCA(MNN)_6_GCA(MNN)_3_- ACA(MNN)_5_GAGACCGCAACGATACGTGCCAC
RevRan_18	CGGCAGTGCCATCTCCGCA(MNN)_6_GCA(MNN)_4_- ACA(MNN)_6_GAGACCGCAACGATACGTGCCAC
RevRan_20	CGGCAGTGCCATCTCCGCA(MNN)_5_GCA(MNN)_5_- ACA(MNN)_8_GAGACCGCAACGATACGTGCCAC
RevRan_23	CGGCAGTGCCATCTCCGCA(MNN)_6_GCA(MNN)_5_- ACA(MNN)_10_GAGACCGCAACGATACGTGCCAC

**Table 2:** Comparison of the efficiency of different elution protocols: solution A: 10 mM Tris pH 7.4, 137 mM NaCl, 1 mM CaCl_2_ and 100 µg/mL Trypsin; solution B: 1.4% Triethylamin in H_2_O pH 10; solution C: 0.2 M glycine-HCl, pH 2.2.

Elution Method		Readout
Solutions Used	Time (min)	Normalized Phage Titer
solution A	30	32×
solution B	10	3×
solution C	10	1×
solution A + solution B	30 + 10	30×
solution B (two times)	10 + 10	18×
solution A (two times)	30 + 20	50×
